# Publisher Correction: Hydroclimatic vulnerability of peat carbon in the central Congo Basin

**DOI:** 10.1038/s41586-022-05539-7

**Published:** 2022-11-15

**Authors:** Yannick Garcin, Enno Schefuß, Greta C. Dargie, Donna Hawthorne, Ian T. Lawson, David Sebag, George E. Biddulph, Bart Crezee, Yannick E. Bocko, Suspense A. Ifo, Y. Emmanuel Mampouya Wenina, Mackline Mbemba, Corneille E. N. Ewango, Ovide Emba, Pierre Bola, Joseph Kanyama Tabu, Genevieve Tyrrell, Dylan M. Young, Ghislain Gassier, Nicholas T. Girkin, Christopher H. Vane, Thierry Adatte, Andy J. Baird, Arnoud Boom, Pauline Gulliver, Paul J. Morris, Susan E. Page, Sofie Sjögersten, Simon L. Lewis

**Affiliations:** 1 Aix Marseille University, CNRS, IRD, INRAE, CEREGE, Aix-en-Provence, France; 2 Institute of Geosciences, University of Potsdam, Potsdam, Germany; 3 MARUM—Center for Marine Environmental Sciences, University of Bremen, Bremen, Germany; 4 School of Geography, University of Leeds, Leeds, UK; 5 School of Geography and Sustainable Development, University of St Andrews, St Andrews, UK; 6 IFP Energies Nouvelles, Earth Sciences and Environmental Technologies Division, Rueil-Malmaison, France; 7 Institute of Earth Surface Dynamics, Geopolis, University of Lausanne, Lausanne, Switzerland; 8 Faculté des Sciences et Techniques, Université Marien Ngouabi, Brazzaville, Republic of the Congo; 9 École Normale Supérieure, Université Marien Ngouabi, Brazzaville, Republic of the Congo; 10 École Normale Supérieure d’Agronomie et de Foresterie, Université Marien Ngouabi, Brazzaville, Republic of the Congo; 11 Faculté de Gestion des Ressources Naturelles Renouvelables, Université de Kisangani, Kisangani, Democratic Republic of the Congo; 12 Faculté des Sciences, Université de Kisangani, Kisangani, Democratic Republic of the Congo; 13 Institut Supérieur Pédagogique de Mbandaka, Mbandaka, Democratic Republic of the Congo; 14 School of Geography, Geology and the Environment, University of Leicester, Leicester, UK; 15 School of Water, Energy and Environment, Cranfield University, Bedford, UK; 16 British Geological Survey, Centre for Environmental Geochemistry, Keyworth, UK; 17 Institute of Earth Sciences, University of Lausanne, Lausanne, Switzerland; 18 NEIF Radiocarbon Laboratory, Scottish Universities Environmental Research Centre (SUERC), Glasgow, UK; 19 School of Biosciences, University of Nottingham, Nottingham, UK; 20 Department of Geography, University College London, London, UK

**Keywords:** Carbon cycle, Palaeoclimate, Tropical ecology

Correction to: *Nature* 10.1038/s41586-022-05389-3 Published online 2 November 2022

This paper was originally published under a standard Springer Nature license (© The Author(s), under exclusive licence to Springer Nature Limited). It is now available as an open-access paper under a Creative Commons Attribution 4.0 International license, © The Author(s). The error has been corrected in the HTML and PDF versions of the article.

