# Supplementary material for: Hydroclimatic vulnerability of peat carbon in the central Congo Basin
Source: Nature. 2022 Nov 2;612(7939):277–82. doi: 10.1038/s41586-022-05389-3 (PMC9729114; doi:10.1038/s41586-022-05389-3)
Supplement: Supplementary file 1 — Reporting Summary [file 41586_2022_5389_MOESM1_ESM.pdf]

nature portfolio

Corresponding author(s): Yannick Garcin

Last updated by author(s): Jul 5, 2022

Reporting Summary

Nature Portfolio wishes to improve the reproducibility of the work that we publish. This form provides structure for consistency and transparency in reporting. For further information on Nature Portfolio policies, see our Editorial Policies and the Editorial Policy Checklist.

Statistics

For all statistical analyses, confirm that the following items are present in the figure legend, table legend, main text, or Methods section.

n/a	Confirmed	
☐	☒	The exact sample size (*n*) for each experimental group/condition, given as a discrete number and unit of measurement
☐	☒	A statement on whether measurements were taken from distinct samples or whether the same sample was measured repeatedly
☐	☒	The statistical test(s) used AND whether they are one- or two-sided<br>*Only common tests should be described solely by name; describe more complex techniques in the Methods section.*
☒	☐	A description of all covariates tested
☒	☐	A description of any assumptions or corrections, such as tests of normality and adjustment for multiple comparisons
☐	☒	A full description of the statistical parameters including central tendency (e.g. means) or other basic estimates (e.g. regression coefficient) AND variation (e.g. standard deviation) or associated estimates of uncertainty (e.g. confidence intervals)
☒	☐	For null hypothesis testing, the test statistic (e.g. *F*, *t*, *r*) with confidence intervals, effect sizes, degrees of freedom and *P* value noted<br>*Give P values as exact values whenever suitable.*
☒	☐	For Bayesian analysis, information on the choice of priors and Markov chain Monte Carlo settings
☒	☐	For hierarchical and complex designs, identification of the appropriate level for tests and full reporting of outcomes
☒	☐	Estimates of effect sizes (e.g. Cohen's *d*, Pearson's *r*), indicating how they were calculated

Our web collection on statistics for biologists contains articles on many of the points above.

Software and code

Policy information about availability of computer code

Data collection	For collection of the hydrogen and carbon isotopes of plant waxes, ISODAT software was used.
Data analysis	The age-depth models were performed using open source rbacon 2.5.7 package running in R 4.1.0 (https://CRAN.R-project.org/package=rbacon). The geospatial analyses and mapping were performed using open source Jupyterhub notebooks (5.7.8; https://jupyter.org/) running Python 3.7.3.

For manuscripts utilizing custom algorithms or software that are central to the research but not yet described in published literature, software must be made available to editors and reviewers. We strongly encourage code deposition in a community repository (e.g. GitHub). See the Nature Portfolio guidelines for submitting code & software for further information.

Data

Policy information about availability of data

All manuscripts must include a data availability statement. This statement should provide the following information, where applicable:

- Accession codes, unique identifiers, or web links for publicly available datasets
- A description of any restrictions on data availability
- For clinical datasets or third party data, please ensure that the statement adheres to our policy

Data that support the findings of this study are available in the PANGAEA repository: https://doi.pangaea.de/10.1594/PANGAEA.938019. Codes for age-depth models and for processing and analysis of geospatial data (climate spaces, tropical peatland distribution and precipitation reconstruction) are available in the IRD Dataverse repository: https://doi.org/10.23708/FO2HGM.

March 2021

Field-specific reporting

Please select the one below that is the best fit for your research. If you are not sure, read the appropriate sections before making your selection.

☐ Life sciences ☐ Behavioural & social sciences ☒ Ecological, evolutionary & environmental sciences

For a reference copy of the document with all sections, see nature.com/documents/nr-reporting-summary-flat.pdf

Ecological, evolutionary & environmental sciences study design

All studies must disclose on these points even when the disclosure is negative.

Study description

This study describes the palaeoenvironmental and palaeohydroclimatic history of the central Congo peatlands, the world's largest tropical peatland complex.

Research sample

This study primarily reports on the analyses of peat cores. Palaeoenvironmental proxies (organic matter properties), preserved pollen and palaeohydrological proxies (hydrogen isotopes of plant waxes) were analysed.

For the geospatial analyses, climatologies were derived from the CHELSA data Version 1.2 publicly available at https://chelsa-climate.org/downloads/ and the current geographical extents of the main peat-bearing tropical regions, including Africa, Southeast Asia/Oceania and America were derived from the PEATMAP dataset publicly available at https://archive.researchdata.leeds.ac.uk/251/.

Sampling strategy

Sampling strategy is described in the Methods section.

Data collection

All data collected were analysed and contributed to the final conclusions.

Timing and spatial scale

Fieldwork and initial data collection occurred during field seasons in the Republic of Congo and in the Democratic Republic of the Congo from 2014 to 2020. All presented analyses were conducted in an intermittent manner from 2017 onwards and were completed in 2021.

Data exclusions

No significant data have been excluded from this study.

Reproducibility

Accuracy and precision of the hydrogen isotopes of plant waxes were controlled by a lab internal n-alkane standard calibrated against the A4-Mix isotope standard (A. Schimmelmann, University of Indiana) every six measurements and by the daily determination of the H3+ factor. Measurement precision was determined by calculating the difference between the analysed values of each standard measurement and the long-term mean of standard measurements, which yielded a 1σ error of <3‰. H3+ factors varied between 4.9 and 5.2 (5.1 ±0.1). Accuracy and precision of the squalane internal standard were both 2‰ (n=238). Precision of the replicate analyses of the n-C27, n-C29 and n-C31 alkanes was 1‰ on average.
Accuracy and precision of the carbon isotopes of plant waxes were determined by measuring n-alkane standards calibrated against the A4-Mix isotope standard every six measurements. The difference between the long-term means and the measured standard values yielded a 1σ error of <0.3‰. Accuracy and precision of the squalane internal standard were both 0.2‰ (n = 264). Precision of the replicate analyses of the n-C27, n-C29 and n-C31 alkanes was 0.1‰ on average.

Randomization

This does not apply to this study as no experiment was conducted.

Blinding

This does not apply to this study as no experiment was conducted.

Did the study involve field work? ☒ Yes ☐ No

Field work, collection and transport

Field conditions

The study sites are in the central Congo Basin. The climate and vegetation are described in the manuscript and it the Methods section.

Location

Cores CEN-17.4 (1° 11' 0.49" N, 17° 38' 23.7" E), EKGKM7-2019 (1° 10' 57.29" N, 17° 48' 18.4" E) and EKG03 (1° 11' 17.7" N, 17° 49' 53.29" E) were collected in a peat-filled interfluvial basin, informally named Ekolongouma, situated between the Likouala-aux-Herbes and Ubangui rivers, in the Likouala Department, Republic of the Congo. Core LOK5-5 (at Lokolama; 0° 19' 36.62" S, 18° 10' 24.37" E) was collected in an interfluvial basin close to the Congo River in the Democratic Republic of the Congo, 177 km from core CEN-17.4, and core BDM1-7 (at Bondamba; 0° 10' 24.38" S, 19° 41' 45.56" E), was collected in a river-influenced valley-floor peatland (not an interfluvial basin) close to the Ruki River, a tributary of the Congo River in Democratic Republic of the Congo, 274 km from core CEN-17.4.

Access & import/export

Fieldwork was permitted by the governments of the Republic of the Congo and of the Democratic Republic of the Congo. The samples were collected and exported with permission of the Republic of the Congo and of the Democratic Republic of the Congo, and imported in the EU under licence.

Disturbance

Minor disturbances occured for few peat sections, which were compacted during transportation, showing a depth reduction of up to 5 cm per 50 cm. The depth of each sample taken was restored on an undistorted depth scale.

Reporting for specific materials, systems and methods

We require information from authors about some types of materials, experimental systems and methods used in many studies. Here, indicate whether each material, system or method listed is relevant to your study. If you are not sure if a list item applies to your research, read the appropriate section before selecting a response.

Materials & experimental systems

n/a	Involved in the study
☒	☐ Antibodies
☒	☐ Eukaryotic cell lines
☒	☐ Palaeontology and archaeology
☒	☐ Animals and other organisms
☒	☐ Human research participants
☒	☐ Clinical data
☒	☐ Dual use research of concern

Methods

n/a	Involved in the study
☒	☐ ChIP-seq
☒	☐ Flow cytometry
☒	☐ MRI-based neuroimaging
